# Bergamot versus beetle: evidence for intraspecific chemical specialization

**DOI:** 10.1093/aobpla/plv132

**Published:** 2015-11-17

**Authors:** Ken Keefover-Ring

**Affiliations:** 1 Department of Ecology and Evolutionary Biology, University of Colorado, N122 Ramaley CB 334, Boulder, CO 80309, USA; 2 Present address: Department of Entomology, University of Wisconsin, 1630 Linden Drive, Madison, WI 53706, USA

**Keywords:** Carvacrol, herbivore specialization, *Monarda fistulosa*, *Physonota unipunctata*, secondary chemical variation, thymol

## Abstract

A large proportion of phytophagous insects show host plant specificity (monophagy or oligophagy), often determined by host secondary chemistry. Yet, even specialists can be negatively affected by host chemistry at high levels or with novel compounds, which may manifest itself if their host species is chemically variable. This study tested for reciprocal effects of a specialist tortoise beetle (*Physonota unipunctata*) feeding on a host plant (*Monarda fistulosa*) with two monoterpene chemotypes [thymol (T) and carvacrol (C)] using a controlled field experiment where larvae fed on caged plants of both chemotypes, haphazardly collected natural plants with and without beetle damage, and growth chamber experiments where larvae that hatched and briefly fed on one chemotype were reared on either chemotype. In the field experiment, plant chemotype did not affect larval weight or length, but did influence larval survival with almost 8.3 % more surviving on T plants. Herbivores reduced seed head area (86.5 % decrease), stem mass (41.2 %) and stem height (21.1 %) of caged plants, but this was independent of host chemotype. Natural plants experienced similar reductions in these variables (74.0, 41.4 and 8.7 %) and T chemotypes were more frequently damaged. In the growth chamber, larval relative growth rate (RGR) differed for both feeding history and year. Larvae from T natal plants reared on T hosts grew at almost twice the rate of those from C and reared on T. Larvae from either T or C natal plants reared on C plants showed intermediate growth rates. Additional analyses revealed natal plant chemotype as the most important factor, with the RGR of larvae from T natal plants almost one-third higher than that of those from C natal plants. These cumulative results demonstrate intraspecific variation in plant resistance that may lead to herbivore specialization on distinct host chemistry, which has implications for the evolutionary trajectory of both the insect and plant species.

## Abstract (precis)

Many plant-eating insects have developed the ability to eat plants that synthesize toxins which they use to defend themselves against herbivores. While these specialized insects are good at dealing with specific plant toxins, plant species with highly variable chemistry can present a challenge. This study tested for reciprocal effects of a specialist tortoise beetle feeding on a host plant with individuals contained two different essential oil toxins. Overall, beetles showed higher survival, growth, and preference for one of the plant's essential oil types. Thus, host plant species with variable chemistry could change the long-term relationship between plant and herbivore.

## Introduction

A large proportion of the millions of insect species eat plants (phytophagous) and a majority of these show high host specificity (monophagous or oligophagous, Jaenike 1990; Ali and Agrawal 2012). In many insect groups, plant chemistry has driven the evolution of this host specialization, with specialists often choosing hosts more on the basis of chemistry than on plant relatedness (Ehrlich and Raven 1964; Jaenike 1990; Rasmann and Agrawal 2011). The specialist strategy has several advantages, including a food source avoided by many other herbivores and the availability of host secondary compounds that many specialist insects use for defence against predators and parasites (Pasteels *et al*. 1983; Weiss 2006). Despite these advantages, even specialists can be negatively affected by the secondary chemistry of their host, especially at high levels (Ali and Agrawal 2012). In addition, novel compounds not usually encountered by specialists probably affect them to a greater or equal extent compared with generalists (Ali and Agrawal 2012).

The ability of specialists to tolerate a limited number of host secondary chemicals may manifest itself if the host species has variable chemistry. Many plant species exhibit large amounts of secondary chemical diversity, often with distinct chemical phenotypes, also known as chemotypes (Vernet *et al*. 1986; Keefover-Ring *et al*. 2009, 2014). Due to structural differences between the compounds found in different chemotypes, herbivore deterrence (Linhart and Thompson 1995, 1999; Ahern and Whitney 2014) and performance (Dray *et al*. 2004; Wheeler 2006; van Leur *et al*. 2008) can vary with chemotype. The few studies that have examined the effects of host plant species with differential chemistry on specialist insect herbivores found varying results. For instance, larval preference and performance of a specialist lepidopteran feeding on two glucosinolate chemotypes of *Barbarea vulgaris* were unaffected by host chemistry (van Leur *et al*. 2008). In contrast, mortality of the specialist Melaleuca snout beetle differed by 64 % (Dray *et al*. 2004) and mass of female adults by 6.3 % (Wheeler 2006) between two terpenoid chemotypes of the invasive weed *Melaleuca quinquenervia*. In addition, the labiate specialist *Arima marginata* showed clear feeding preferences for different *Thymus vulgaris* chemotypes, consuming higher amounts of foliage containing non-phenolic monoterpenes and avoiding phenolic chemotypes (Linhart and Thompson 1999). These patterns of differential performance and survival of a specialist herbivore on a host with variable chemistry have important implications for the evolutionary trajectory of both insect and plant.

Many species in the beetle family Chrysomelidae specialize on plants high in secondary metabolites (Pasteels *et al*. 1983; Becerra *et al*. 2001), including the subfamily Cassidinae, known as tortoise beetles due to the shell-like appearance of the adult. Cassidine adults and larvae often eat the same host plant, and larvae of many species feed gregariously for at least some part of their development (Chaboo *et al*. 2014). The larvae of some tortoise beetle species accumulate a ‘fecal shield’ on fork-like paired urogomphi located on the last segment of the abdomen, which consists of frass, sometimes combined with exuvia, and is often rich in host plant secondary compounds (Gómez *et al*. 1999; Vencl *et al*. 1999; Keefover-Ring 2013). Fecal shields have been shown to be an effective defence against predators (Olmstead and Denno 1993; Gómez *et al*. 1999; Vencl *et al*. 1999, 2005; Eisner and Eisner 2000).

The tortoise beetle *Physonota unipunctata* uses wild bergamot, *Monarda fistulosa* (Lamiaceae), as its only host (Hamilton 1884; Criddle 1926; Sanderson 1948). Like most labiates, *M. fistulosa* produces essential oils (mono- and sesquiterpenes) in glandular peltate trichomes on leaves and reproductive tissue (petals and calyces) surfaces (Heinrich 1973). Throughout most of its range, *M. fistulosa* plants are one of two chemotypes, producing one of two different phenolic monoterpenes as their major signature monoterpene: thymol (T; 2-isopropyl-5-methylphenol) or carvacrol (C; 5-isopropyl-2-methylphenol) (Scora 1967; Weaver *et al*. 1995; Johnson *et al*. 1998; Keefover-Ring 2013). While these two compounds deter a variety of pathogens and parasites more than other non-phenolic monoterpenes (Linhart and Thompson 1995, 1999), *P. unipunctata* feeds on plants expressing both chemotypes (Keefover-Ring 2013). Despite their structural similarity, however, C chemotypes had greater overall toxicity than T to a variety of herbivores feeding on common thyme, another labiate species with both chemotypes (Linhart and Thompson 1999). These previous studies suggest that there may be differences in the performance of a specialist herbivore on these two *M. fistulosa* chemotypes with concomitant effects on plant fitness.

This study examined whether polymorphic host chemistry differentially affects preference and performance of a monophagous herbivore and, in turn, whether the observed differences influence patterns of herbivory. The study used a manipulative field experiment with plants of the two chemotypes, subjected to controlled levels of herbivory, to assess the effects of larval herbivory on *M. fistulosa* growth and fitness and *P. unipunctata* larval growth and survival. Also in the field, stems were collected from non-experimental plants with natural herbivory and controls with no damage to see whether beetles showed a chemotype preference. Finally, to determine possible differences in larval performance (relative growth rate (RGR)) due to host chemistry, broods of larvae that had initially hatched and briefly fed on one of the two common chemotypes were equally divided and reared on foliage from plants of either chemotype in a growth chamber.

## Methods

### Study organisms

*Monarda fistulosa* L. var. *menthifolia* (Lamiaceae), commonly known as wild bergamot, bee balm or horse mint, is a perennial mint that occurs in most of the continental USA (expect for Alaska, California and Florida), all of the southern Canadian provinces as far east as Quebec and in the Northwest Territory (Straley 1986; USDA 2008). Individual plants consist of multiple stems that arise directly from the ground ∼0.6 to >1 m high, each with one to a few terminal capitate inflorescences with lavender tubular flowers, subtended by leaf-like bracts. Below the capitula are alternating pairs of lanceolate leaves with serrated margins. Two common chemotypes of *M. fistulosa* occur in Colorado, producing one of two phenolic monoterpenes, thymol or carvacrol, as the primary compound in their essential oil (K. Keefover-Ring, unpubl. data).

*Monarda fistulosa* has few herbivores (Davis *et al*. 1988; Wyckhuys *et al*. 2007); however, the one-spotted tortoise beetle *P. unipunctata* (Coleoptera: Chrysomelidae) uses this species as its only host for both larvae and adults (Hamilton 1884; Criddle 1926; Sanderson 1948). In Colorado, adults lay a cluster of eggs covered by a filamentous film on the underside of a leaf in mid-May. After hatching, larvae chew their way through to the top of the leaf and usually climb the plant stem as a group to feed on higher leaves. Early instar larvae feed gregariously, often on the underside of leaves, and maintain a large fecal shield on a fork-like structure located on the last segment of the abdomen, in which they concentrate host compounds (Keefover-Ring 2013). Larvae hold the shields above their bodies and, when disturbed, wave the shield at the offender (K. Keefover-Ring, pers. obs.).

### Selection and chemotype determination of field plants

In early June 2005, I haphazardly chose 122 individual multi-stemmed *M. fistulosa* plants at a site designated Chicken Ranch Gulch, located on Flagstaff Mountain in the foothills ∼2 km west of Boulder, CO (39°50.817′N, 105°18.467′W) and marked them with bamboo poles and numbered tags. Since *Monarda fistulosa* spreads vegetatively, plants were selected at least two 2 m apart to ensure they represented separate individuals.

To determine the chemotype of an individual plant, I analysed a single leaf clipped from each individual. Each leaf sample was carefully rolled to fit to the bottom of a 2-mL microcentrifuge tube and then completely submerged with 1.00 mL of an internal standard solution (0.1 mg mL^−1^ *m*-xylene in pure ethanol), sonicated in a ambient temperature water bath for 1 min and then briefly vortex mixed. Samples were left to extract for 7 days at ambient temperature and the resulting solutions analysed by gas chromatography (GC) with flame ionization detection by a previously described method (Keefover-Ring 2013). I then assigned plant chemotypes based on the monoterpene that constituted the highest percentage of a plant's essential oil. There were two such chemotypes, thymol and carvacrol, with either T or C as their respective dominant monoterpene. Other work has shown that, except for their dominant terpene, these two chemotypes have comparable chemical profiles of numerous other monoterpenes and similar amounts of their main and total monoterpenes (Keefover-Ring 2013). The GC analyses identified 103 plants as T and 19 as C (see Results) chemotypes, from which I chose 44 T plants and 19 C plants for use in the experiment described below.

### Field experiments examining how host chemotype mediates host–herbivore interactions

To test whether host chemotype affects beetle larvae performance and survival and in turn plant size and fitness, I conducted a field experiment involving controlled larval feeding on caged plants of both T and C chemotypes and also randomly collected ‘natural’ plants of unknown chemotype with and without herbivore damage. For the controlled experiment with caged plants, in mid-June 2005, I randomly assigned 63 of the chemotyped plants at the Chicken Ranch Gulch site to either a no-herbivory (T plants, *n* = 21; C plants, *n* = 8) or herbivory (T plants, *n* = 23; C plants, *n* = 11) treatment. This time of year corresponds to when larvae are just beginning to hatch and feed at the site. For the herbivory treatment, I collected first instar *P. unipunctata* larvae from plants of unknown chemotype found within 10 m of the focal plants. These larvae were placed in a container and gently but thoroughly mixed to minimize any natal plant effects. I then enclosed a single stem of each of the chosen experimental plants with a cage and, before closing, transferred 10 larvae together onto an upper leaf. The number of larvae used was based on earlier observations of single stems from 30 plants at the site, which found densities of 12.5 (SD = 6.4) first instar larvae per stem. I used a lower number, since larvae would not be able to move from caged stems. Cages consisted of a forest green fine mesh sewn into a tube, maintained in a cylinder shape with upper and lower wire loops, which was gathered and tied with a thin wire at the base of the stem. Two bamboo poles, stuck into the ground and attached to the wire loops, supported the cages. The no-herbivory treatment consisted of separate plants with a single stem enclosed in identical cages, but with no larvae added.

On 28 and 29 June 2005, I removed the cages, collected all surviving larvae and brought them to the lab for weight (to the nearest 0.1 mg) and length measurements (to the nearest 0.5 mm from mandibles to tail fork) to look for possible effects of host chemotype on larvae. Using the PROC GLM function in SAS version 9.1 (SAS Institute 2013), I tested whether plant chemotype (T vs. C) affected the survival or performance (length and weight) of larvae by running separate paired *t*-tests using the mean values of these three variables (survival, length and weight) from all larvae on a single plant. All experimental plants were left in the field to allow natural flowering, pollination, seed set and maturation. Plants were eventually harvested between 19 and 26 August 2005. Harvesting involved clipping caged ramet stems from individual plants, subjected to either no herbivory or herbivory, at ground level and placing them in separate paper bags. In addition, to assess ‘natural’ levels of herbivory, I also haphazardly collected stems from surrounding non-experimental plants that had obviously been subject to either no herbivory (*n* = 46) or herbivory (*n* = 41), and determined their chemotypes as above. All stems were then allowed to air-dry for 1 week and then weighed to the nearest 0.01 g, their total length measured to the nearest 0.1 cm and the area of their seed head calculated (mm^2^, calculated as *π* times the product of half of two orthogonal measurements of the shortest and longest diameters). I used seed head area as a proxy for seed number and hence plant fitness, since a correlation analysis between seed number and seed head area for 25 randomly chosen stems showed a strong positive relationship (*r* = 0.79, *P* < 0.001).

To test whether the effects of herbivores on the plants varied with chemotype (T vs. C), I used the PROC GLM function to perform separate two-factor analyses of variance (ANOVAs), with chemotype and herbivory treatment as the two factors, for seed head area, stem mass and stem height using only the caged stems with herbivores present. I could not do a chemotype comparison with the natural collection plants, since only a single C plant was present in the natural herbivore-damaged plants.

To compare the levels of herbivory between experimental and natural plants, irrespective of chemistry, I also tested for differences in seed head area, stem mass and height among four different treatments: stems in experimental cages without (i) and with (ii) herbivores, and natural undamaged (iii) and herbivore-damaged (iv) plants. I used the PROC GLM function to run separate ANOVAs for each variable among the four treatments and then tested all pair-wise comparisons with a Tukey *post hoc* test.

Finally, using the PROC FREQ function, I performed separate goodness-of-fit tests on the natural undamaged and herbivore-damaged plants to determine whether either set differed from the expected chemotype ratio of the site. I calculated the expected chemotype ratio using the 122 plants initially analysed (103 T : 19 C). Thus, the chemotype ratio of any subsequently collected set of plants should be ∼84 % T : 16 % C.

### Growth chamber larval performance experiments

I conducted feeding trials in growth chambers to determine the performance of *P. unipunctata* larvae on the two *M. fistulosa* chemotypes in 2005 and 2006. In both years, I collected single plant stems from natal plants with either egg masses or newly hatched (1–2 days old) first instar larvae from two locations on the Colorado Front Range: Chicken Ranch Gulch and a site just south of the former Rocky Flats weapons site (39°54.783′N, 105°1.167′W). I immediately placed hatched first instar larvae into the experimental treatments, but left egg masses intact on the natal host plant cuttings. The stems with egg masses were kept in floral water picks until larvae hatched and chewed through the leaf, at which time these first instar larvae were incorporated into the feeding trials. I divided broods, which averaged ∼10 individuals, as evenly as possible, and randomly placed the groups into one of two feeding treatments, where they ate either T or C chemotype foliage only. The foliage used for food in the experiment consisted of single stems of *M. fistulosa* cut from several plants near the Gregory Canyon trailhead, ∼2 km east of the Chicken Ranch Gulch site, where plants of both chemotypes occur (K. Keefover-Ring, unpubl. data). I used a single leaf from all plants from which eggs or larvae were collected (natal plants) and all food plants to determine the chemotype of each by GC. I then took ∼12 cm cuttings of either T or C foliage, placed the stem in small holes punched in the centre of screw-top lids of 100 mL plastic cups filled with fresh water and put the cups in upright 6.5 × 12.5 × 17.5 cm clear plastic containers that had a section of plastic mesh on the front for air exchange. Before I transferred larvae to the foliage, I counted and weighed all individuals of a group together to the nearest 0.01 g, first removing any fecal shields. I kept all feeding trial containers in a growth chamber (Percival Scientific, Perry, IA, USA) maintained at 25 °C, with a light regime of 14 h light and 10 h dark. After larvae entered their final instar, but before pupation, I took them from their host plants, removed any fecal shields, and counted and obtained the live weight of the entire group. I calculated RGR as the difference between the initial and final mean larval weights, divided by the initial mean larval weight, divided by time (mg mg^−1^ day^−1^) (Bowers *et al*. 1991).

To test for differences in larval performance due to feeding history (natal and host plant chemistry), I used PROC GLM with the TEST option in the RANDOM statement to perform a mixed-model two-factor ANOVA with a treatment called delta (*δ*) as a fixed factor and year and the interaction of *δ* and year as random factors, using larval RGR as the dependent variable. The *δ* treatment consisted of four groups: the combination of the chemotype of the natal plant from which a larva was initially collected and the chemotype of the host plant they were reared upon (T → T, T → C, C → C and C → T). I made all pair-wise comparisons of these four treatments using least squared means with a Tukey–Kramer adjustment. Also, since I initially thought that the chemotype of either natal or host plant would be important, I employed *a priori* contrast codes to test for differences between the two chemotypes separately for both natal and host plant.

## Results

### Field experiment to examine reciprocal effects of host chemotype

#### Effects on larval performance and survival

**Figure 1.**
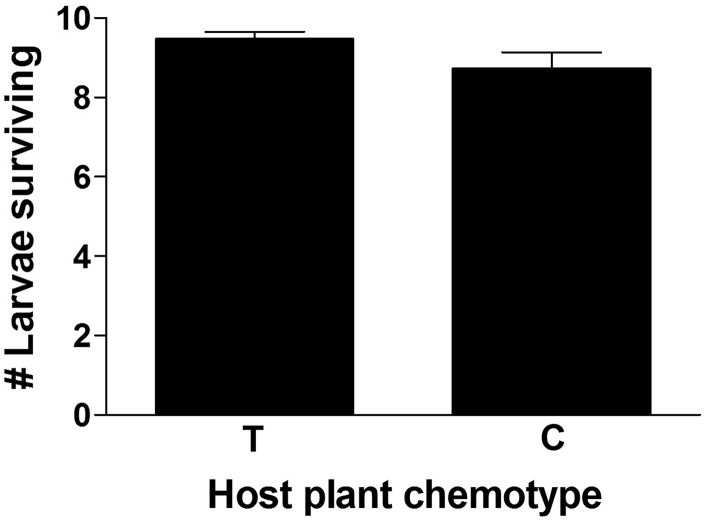
Mean (±1 SE) number of *P. unipunctata* larvae surviving after being caged on *M. fistulosa* stems of two different chemotypes in the field. T, thymol chemotype; C, carvacrol chemotype.

Host plant chemotype (T or C) did not affect larval weight (*F*_1, 32_ = 1.0, *P* = 0.333) or length (*F*_1, 32_ = 0.8, *P* = 0.563). However, host chemotype did marginally influence larval survival (*F*_1, 32_ = 4.0, *P* = 0.055), with ∼8.3 % more surviving on T plants (Fig. 1).

#### Effects on host plant size and fitness

The separate two-factor ANOVAs (using the experimental caged stems only), testing for differences in *M. fistulosa* seed head area, stem mass and stem height for the factors of chemotype and herbivory, showed no differences due to chemotype, large differences with herbivory and no interaction between these two factors for any of the three variables (Table 1).

**Table 1.** *F* and *P* values from the two-factor ANOVAs assessing the effect of chemotype (Chemo; T or C), herbivory (Herb; without or with) and their interactions on *M. fistulosa* seed head area, stem mass and height of caged stems. Boldface *P*-values indicate significance at *α* = 0.05.

Source	df	Seed head area		Stem mass		Stem height	
		*F*	*P*	*F*	*P*	*F*	*P*
Chemotype	1	0.2	0.640	0.1	0.782	0.0	0.928
Herbivory	1	19.0	**<0.001**	27.8	**<0.001**	18.5	**<0.001**
Chemo × Herb	1	0.1	0.727	0.0	0.850	0.1	0.814

**Figure 2.**
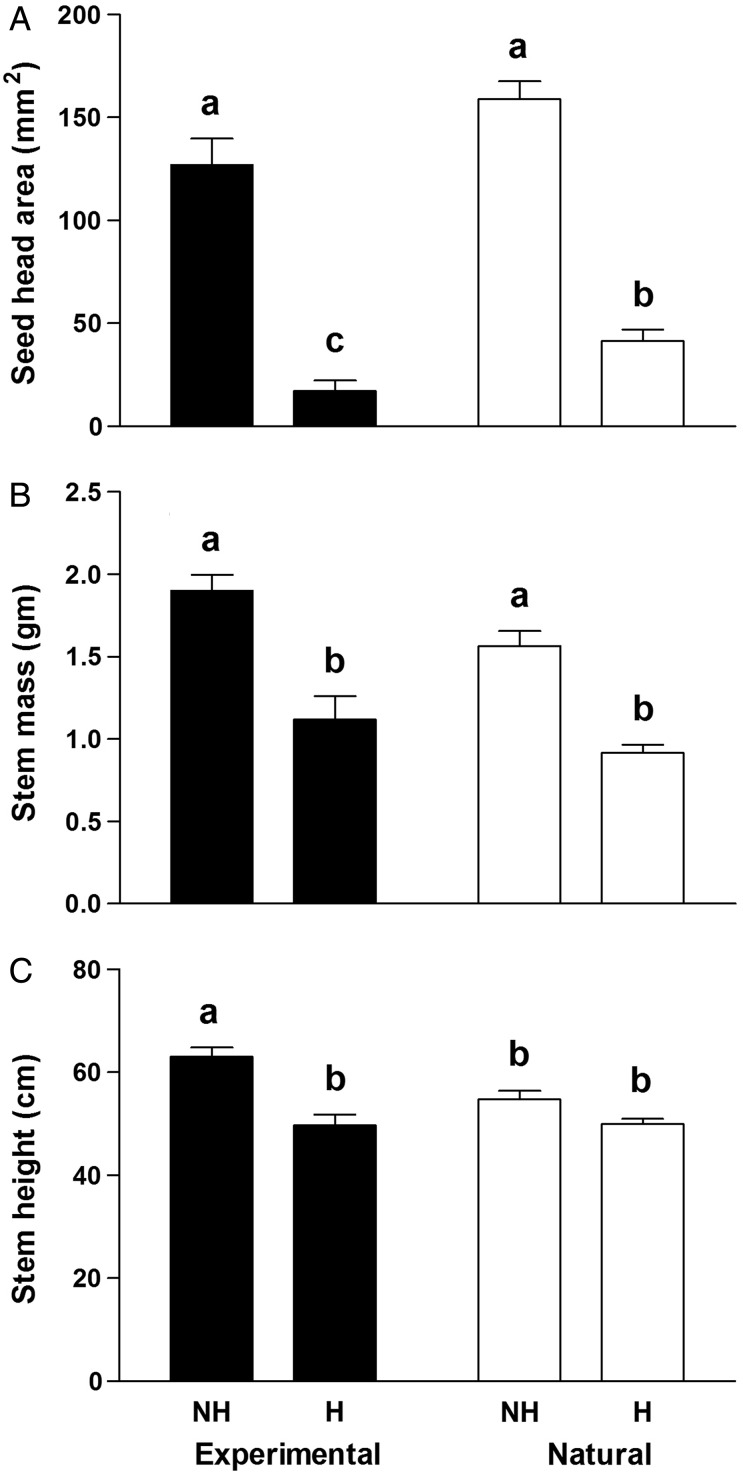
Mean (±1 SE) values for seed head area (A), stem mass (B) and stem height (C) of *M. fistulosa* with and without *P. unipunctata* larvae present under both experimental and natural conditions in the field. NH, no herbivores; H, herbivores present. ‘Experimental’ refers to plants included in the cage experiment and ‘Natural’ refers to stems haphazardly collected from plants with and without natural herbivore damage.

The separate ANOVAs, comparing experimental caged plants with naturally collected stems for herbivory, irrespective of chemistry, showed considerable differences for these three variables among the four treatments [stems in cages without (i) and with (ii) herbivores, and natural undamaged (iii) and herbivore-damaged (iv) plants] (Fig. 2). Although experimental and natural stems without larvae did not differ in seed head size, herbivory greatly reduced seed head area for both (Fig. 2A; *F*_3, 147_ = 79.1, *P* < 0.001). Experimental and natural stems without larvae had 7.4 and 3.8 times greater seed head areas, respectively, compared with their experimental counterparts with larvae. In addition, natural stems with herbivores had more than twice the seed head area of caged stems with larvae. While experimental stems without herbivores had more mass than natural stems of the same herbivore treatment, herbivory caused reductions in mass of ∼41 % for experimental and natural stems, compared with their respective uneaten stems (Fig. 2B; *F*_3, 147_ = 27.6, *P* < 0.001). Experimental caged stems without herbivores attained greater heights than the other treatments, and were 13 % taller than natural stems with larvae and ∼21 % greater in height than both the caged and natural stems subjected to tortoise beetle larvae (Fig. 2C; *F*_3, 147_ = 12.1, *P* < 0.001).

### Natural plant chemotypes and herbivory

Out of the 46 natural haphazardly collected plants without tortoise beetle damage, 41 belonged to the T chemotype and 5 to the C. A goodness-of-fit test showed that this chemotype ratio did not differ from the chemotype ratio expected for the site (*χ*^2^ = 0.8, *P* = 0.38). In contrast, the 41 natural herbivore-damaged plants included only one C chemotype, a ratio that departed from expected (*χ*^2^ = 5.4, *P* = 0.02).

### 2005 and 2006 larval performance experiments

**Figure 3.**
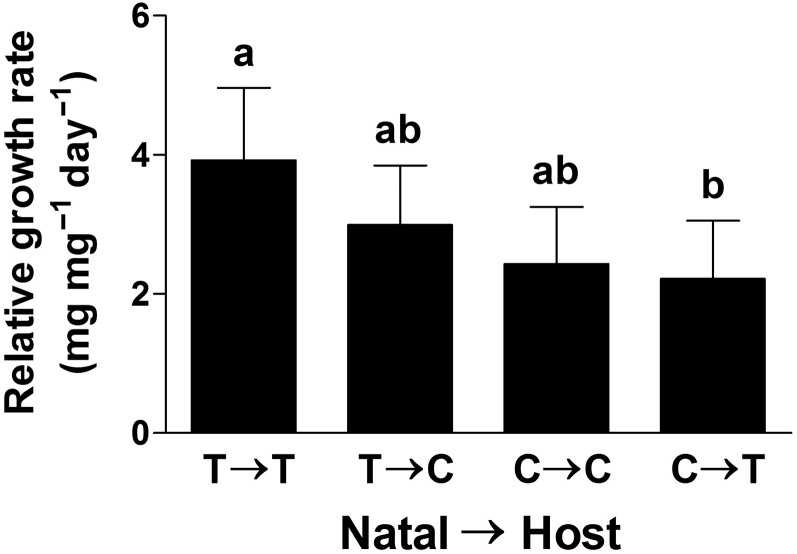
Mean (±1 SE) RGR of *P. unipunctata* larvae from the four treatment combinations: natal = *M. fistulosa* plant chemotype that a group of larvae originated from (letter before the arrow); host = plant chemotype that a group of larvae were reared on (letter after the arrow). T, thymol chemotype; C, carvacrol chemotype. Bars with the same letters did not differ. See Table 2 for corresponding statistical results.

**Figure 4.**
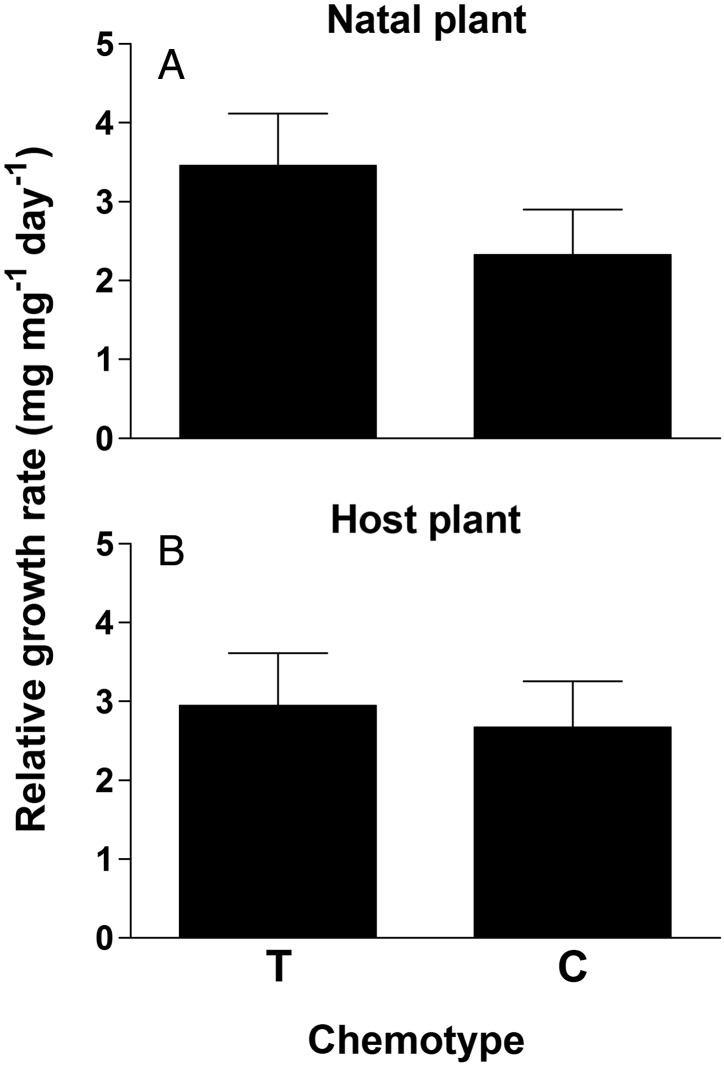
Mean (±1 SE) RGR of *P. unipunctata* larvae depending on either the chemotype of the natal plant they originated on (A) or the chemotype of the host plants they were reared on (B). T, thymol chemotype; C, carvacrol chemotype. See Table 2 for corresponding statistical results.

The mixed-model two-factor ANOVA for larvae RGR differed for both year and *δ*, but these factors did not interact, indicating similar patterns for both years (Table 2). Larvae in the 2005 feeding trails had a RGR (RGR ± SE; 4.2 ± 0.7 mg mg^−1^ day^−1^) more than twice that seen in 2006 (1.8 ± 0.2 mg mg^−1^ day^−1^). In addition to the differences in year, the four levels of the *δ* treatment also showed differences. Specifically, larvae that were taken from T chemotype natal plants and reared on T host plants had the highest growth rates, which were almost twice the rate of larvae taken from C and reared on T chemotype plants (Fig. 3, Table 2). Larvae from either T or C natal plants reared on C host plants performed at intermediate levels. Results of the contrast analyses revealed that the most important factor in larval performance was the chemotype identity of the natal plant, with a difference among chemotypes for natal plants (Fig. 4A, Table 2) but not for host plants (Fig. 4B, Table 2). Overall, larvae from eggs oviposited and emerging on T plants demonstrated a RGR almost one-third higher than if they originated from a C natal plant (Fig. 4A).

**Table 2.** Results of the mixed-model two-factor ANOVA and contrast analyses of the RGR of *P. unipunctata* larvae from natal *M. fistulosa* plants of either T or C chemotype reared on host plants of either T or C chemotype (*δ*) in a growth chamber. Boldface *P*-values indicate significance at *α* = 0.05.

Source of variation	RGR (mg mg^−1^ day^−1^)		
	df	*F*	*P*
Year	1, 34	52.4	**0.003**
*δ*	3, 34	12.7	**0.033**
Year × *δ*	3, 34	0.3	0.854
Contrast analyses results			
Natal chemotype	1, 34	9.2	**0.005**
Host chemotype	1, 34	0.0	0.902

## Discussion

The results of this work demonstrate that the relationship between a specialist insect and its host plant can have more complex ramifications than a simple exploitation of host by herbivore. While the reduction in fitness that *M. fistulosa* sustained as a result of herbivory was clearly evident and beetles showed preference for one chemotype, there were also host effects on *P. unipunctata* larval survival and performance, which differed as a function of host chemistry. I begin with a discussion on how beetle herbivory affected host plant fitness, then look at the reciprocal effects of the host on its herbivore and finish with the implications of the results for chemical polymorphism in *M. fistulosa*.

### Herbivore effects on host plant

The field experiment portion of this study showed that, while levels of herbivory did not differ due to plant chemistry (no differences in herbivory between T and C caged plants in the field), *P. unipunctata* larvae greatly reduced seed head area, mass and height of *M. fistulosa* stems. The lack of a difference in herbivory between chemotypes may have resulted from collecting and mixing larvae from multiple non-experimental plants of unknown chemotype, which may have significantly reduced the strong effect of natal host chemistry observed in the feeding trials. In addition, while both experimental caged plants with larvae and plants with natural herbivory had much smaller seed heads than controls, caged plants received slightly more damage. The stocking levels of larvae in cages (10 per plant) may account for this. Random observations of other single stems at the site with herbivores present, conducted near the end of the manipulative experiment, found that although early instar larvae initially feed in larger groups (up to ∼18 per stem), the number of later instars feeding together was much smaller (2–4 per stem). Lower larval density at later instars was noted in another tortoise beetle species (Costa *et al*. 2007). Since individual *M. fistulosa* plants have multiple stems, often with leaves in contact, *P. unipunctata* larvae can easily move to other stems on the plant to reduce localized feeding density, an activity not possible with cages. Migration to other host plants seems unlikely, given the observed separation of individual plants in the field.

In contrast to seed set, herbivory affected stem mass and height less; however, these differences would likely also affect future plant performance. In general, stems without larvae had close to twice the mass and were slightly taller than those with herbivory. Aboveground biomass has been shown to correlate with total plant fitness in other studies (Hartnett 1990; Schmid and Weiner 1993) and flowering stem height is an important factor for plant reproduction, with taller stalks receiving more pollinator visits, resulting in higher rates of outcrossing (Gómez 2003; Carromero and Hamrick 2005).

While chemotype-mediated effects of herbivores were not apparent in the manipulated caged plants, the higher rates of herbivory seen on T chemotypes in natural plants indicated that *P. unipunctata* exerts more pressure on this chemotype, driven perhaps by the apparent lower toxicity of T. This leads to questions about whether host secondary chemistry influences adult oviposition decisions, which ultimately dictates the chemotype that larvae feed on. Wheeler and Ordung (2005) found that females of the biocontrol psyllid insect *Boreioglycaspis melaleucae*, used against invasive *M. quinquenervia*, oviposited more than twice as many eggs on the leaves of one of the plant's two sesquiterpene chemotypes. Whether *P. unipunctata* adults use the variable terpene chemistry of *M. fistulosa* as oviposition cues is not known. Given the results of the performance experiments, it would certainly be more advantageous to leave eggs on a T chemotype host.

### Host plant effects on herbivores

Both the field and growth chamber experiments showed that host plant chemistry differentially affected tortoise beetle larvae. First, larvae that fed on T chemotype plants in the field had >8 % higher survival. Next, in the growth chamber feeding trials, larval performance also differed with plant chemistry, manifested by increased growth rates of larvae from T chemotype natal plants. These results are interesting given the chemical similarities between the dominant monoterpenes in the two chemotypes: thymol and carvacrol. Structurally related phenolic isomers (aromatic rings with hydroxyl side groups differing by a single carbon position), both compounds generally deter various animals and plants more than other non-phenolic monoterpenes, but C chemotype plants consistently shows greater activity (Linhart and Thompson 1995, 1999). This is not surprising, since even closely related molecules can vary in their toxicity due to enzyme specificity (Stipanovic *et al*. 2005). Other studies have demonstrated differential insect performance or feeding on intraspecific chemotypes with small chemical differences in their main constituents. van Leur *et al*. (2008) found that two glucosinolate phenotypes of the crucifer *B. vulgaris* affected specialist and generalist lepidopterans differently, even though the main compounds differed by the presence or absence of a single hydroxyl group. Also, Ahern and Whitney (2014) showed that grasshoppers ate consistently less plant material containing *cis*-fused sesquiterpene lactones, compared with *trans*-fused stereoisomers.

The reasons for differential performance of *P. unipunctata* on *M. fistulosa* chemotypes may involve more than just toxicity differences between T and C, highlighted by the significant results for chemotype switching (natal → host). This indicates that either adult or larval feeding experience, possibly combined with inherent compound toxicity, is important in determining future larval performance. Given the feeding trial patterns observed in this study, it is possible that a larva's initial exposure to specific host chemistry may determine its ability to detoxify subsequent novel compounds. Nagasawa and Matsuda (2005) found that feeding experience was important with another tortoise beetle, *Cassida nebulosa*, which normally feeds on chenopodiaceous weeds. Adults only weakly accepted a closely related food plant (spinach) if initially reared on their normal host *Chenopodium album*. Naive adults, however, readily fed on both plants within 24 h. Finally, another study found patterns similar to the feeding trial results of the current work. Male parsnip web worms from parents collected on the high furanocoumarin-producing cow parsnip that were also fed cow parsnip suffered the highest mortality, compared with those from parents collected from the less toxic wild parsnip and fed wild parsnip (Berenbaum and Zangerl 1991). Survivorship was intermediate with the two other treatment combinations.

## Conclusions

Intraspecific chemical specialization of an herbivore has several possible outcomes for the evolutionary trajectory of both the plant and insect species. Herbivore chemotype preference could lead to shifts in local chemotype frequencies, eventually resulting in populations with more well-defended plants. In this study, the combined results of higher number of larvae surviving on T chemotypes in the field, higher natural rates of herbivory on T plants and the better performance of larvae from T natal plants indicated that tortoise beetles had clear chemical preferences that could affect *M. fistulosa*'s chemical polymorphism. Adults may use host volatiles of preferred chemotypes to further strengthen the association, since it has been shown that herbivorous insects can use emitted terpenoids to find their host (Zebelo *et al*. 2011) and that *M. fistulosa* produces large amounts of volatiles, both in the absence and presence of herbivores (Keefover-Ring 2013). The ability to find a host with specific chemistry, combined with increased performance and survival and reinforced by chemotype-specific oviposition preferences (Wheeler and Ordung 2005), could lead *P. unipunctata* to become an even narrower specialist on a single chemotype. Given the ratio of chemotypes observed at the site (predominately more of the preferred T plants), however, it seems likely that multiple selective forces, some in opposing directions, may work to maintain the genetic polymorphism, as has been proposed for essential oil variation in *T. vulgaris* (Linhart and Thompson 1999). For example, since tortoise beetle larvae are known to use plant secondary chemistry for defensive purposes (Nogueira-de-Sá and Trigo 2005; Vencl *et al*. 2005), and *P. unipunctata* concentrates phenolic host terpenes in its fecal shields (Keefover-Ring 2013), a trade-off could exist between the negative effects (reductions in growth rate or survival) of a more toxic host and the positive effects of carrying a fecal shield with more deterrent compounds. Vencl *et al*. (2005) showed that while specialist tortoise beetle larvae have to deal with more toxic hosts than generalists, they were better at exploiting host chemistry for defensive purposes.

The relationship between specialist insects, even those considered completely monogamous, and their host plants may be more complex than initially thought. A better awareness of the chemical diversity present in many plant species, combined with how various compounds may differentially affect or be used by herbivores, can help us predict intraspecific chemical specialization and ultimately refine our ideas about the evolution of herbivore feeding strategies.

## Sources of Funding

A Walker Van Riper grant from the University of Colorado Museum of Natural History and a Graduate Student Fellowship from the Department of Ecology and Evolutionary Biology at University of Colorado Boulder to the author partially supported this research.

## Contributions by the Authors

The author was the sole contributor to the presented research.

## Conflict of Interest Statement

None declared.

## References

[PLV132C1] Ahern JR, Whitney KD 2014 Stereochemistry affects sesquiterpene lactone bioactivity against an herbivorous grasshopper. Chemoecology 24:35–39. 10.1007/s00049-013-0144-z

[PLV132C2] Ali JG, Agrawal AA 2012 Specialist versus generalist insect herbivores and plant defense. Trends in Plant Science 17:293–302. 10.1016/j.tplants.2012.02.006 22425020

[PLV132C3] Becerra JX, Venable DL, Evans PH, Bowers WS 2001 Interactions between chemical and mechanical defenses in the plant genus *Bursera* and their implications for herbivores. American Zoologist 41:865–876.

[PLV132C4] Berenbaum MR, Zangerl AR 1991 Acquisition of a native hostplant by an introduced oligophagous herbivore. Oikos 62:153–159. 10.2307/3545260

[PLV132C5] Bowers MD, Stamp NE, Fajer ED 1991 Factors affecting calculation of nutritional induces for foliage-fed insects: an experimental approach. Entomologia Experimentalis et Applicata 61:101–116. 10.1111/j.1570-7458.1991.tb02402.x

[PLV132C6] Carromero W, Hamrick JL 2005 The mating system of *Verbascum thapsus* (Scrophulariaceae): the effect of plant height. International Journal of Plant Sciences 166:979–983. 10.1086/449315

[PLV132C7] Chaboo CS, Frieiro-Costa FA, Gómez-Zurita J, Westerduijn R 2014 Origins and diversification of subsociality in leaf beetles (Coleoptera: Chrysomelidae: Cassidinae: Chrysomelinae). Journal of Natural History 48:2325–2367. 10.1080/00222933.2014.909060

[PLV132C8] Costa JF, Cosio W, Gianoli E 2007 Group size in a gregarious tortoise beetle: patterns of oviposition vs. larval behaviour. Entomologia Experimentalis et Applicata 125:165–169. 10.1111/j.1570-7458.2007.00612.x

[PLV132C9] Criddle N 1926 A note on the synonymy of certain species of *Physonota* (Coleoptera). The Canadian Entomologist 58:207–208. 10.4039/Ent58207-8

[PLV132C10] Davis MA, Lemon KM, Dybvig A 1988 The impact of prescribed burning and herbivorous insects on seed production and viability in two prairie forbs. Paper presented at the prairie: roots of our culture; foundation of our economy: Proceedings of the Tenth North American Prairie Conference of Texas Women's University, Denton, TX.

[PLV132C11] Dray FA, Bennett BC, Center TD, Wheeler GS, Madeira PT 2004 Genetic variation in *Melaleuca quinquenervia* affects the biocontrol agent *Oxyops vitiosa*. Weed Technology 18:1400–1402. 10.1614/0890-037X(2004)018[1400:GVIMQA]2.0.CO;2

[PLV132C12] Ehrlich PR, Raven PH 1964 Butterflies and plants: a study in coevolution. Evolution 18:586–608. 10.2307/2406212

[PLV132C13] Eisner T, Eisner M 2000 Defensive use of a fecal thatch by a beetle larva (*Hemisphaerota cyanea*). Proceedings of the National Academy of Sciences of the USA 97:2632–2636. 10.1073/pnas.050002197 10681467 PMC15980

[PLV132C14] Gómez JM 2003 Herbivory reduces the strength of pollinator-mediated selection in the Mediterranean herb *Erysimum mediohispanicum*: consequences for plant specialization. The American Naturalist 162:242–256. 10.1086/376574 12858267

[PLV132C15] Gómez NE, Witte L, Hartmann T 1999 Chemical defense in larval tortoise beetles: essential oil composition of fecal shields of *Eurypedus nigrosignata* and foliage of its host plant, *Cordia curassavica*. Journal of Chemical Ecology 25:1007–1027. 10.1023/A:1020821507014

[PLV132C16] Hamilton J 1884 On *Trogoderma ornata*, *Physonota unipunctata* and *Tanysphyrus lemnæ*. The Canadian Entomologist 16:133–136. 10.4039/Ent16133-7

[PLV132C17] Hartnett DC 1990 Size-dependent allocation to sexual and vegetative reproduction in four clonal composites. Oecologia 84:254–259. 10.1007/BF00318281 28312762

[PLV132C18] Heinrich G 1973 [Development, fine structure and oil content of gland hairs from *Monarda fistulosa*]. Planta Medica 23:154–166 (in German). 4705795 10.1055/s-0028-1099427

[PLV132C19] Jaenike J 1990 Host specialization in phytophagous insects. Annual Review of Ecology and Systematics 21:243–273. 10.1146/annurev.es.21.110190.001331

[PLV132C20] Johnson HA, Rogers LL, Alkire ML, McCloud TG, Mclaughlin JL 1998 Bioactive monoterpenes from *Monarda fistulosa* (Lamiaceae). Natural Product Letters 11:241–250. 10.1080/10575639808044955

[PLV132C21] Keefover-Ring K 2013 Making scents of defense: do fecal shields and herbivore-caused volatiles match host plant chemical profiles? Chemoecology 23:1–11. 10.1007/s00049-012-0117-7

[PLV132C22] Keefover-Ring K, Thompson JD, Linhart YB 2009 Beyond six scents: defining a seventh *Thymus vulgaris* chemotype new to southern France by ethanol extraction. Flavour and Fragrance Journal 24:117–122. 10.1002/ffj.1921

[PLV132C23] Keefover-Ring K, Ahnlund M, Abreu IN, Jansson S, Moritz T, Albrectsen BR 2014 No evidence of geographical structure of salicinoid chemotypes within *Populus tremula*. PLoS ONE 9:e107189 10.1371/journal.pone.0107189 25299342 PMC4191948

[PLV132C24] Linhart YB, Thompson JD 1995 Terpene-based selective herbivory by *Helix aspersa* (Mollusca) on *Thymus vulgaris* (Labiatae). Oecologia 102:126–132. 10.1007/BF00333320 28306817

[PLV132C25] Linhart YB, Thompson JD 1999 Thyme is of the essence: biochemical polymorphism and multi-species deterrence. Evolutionary Ecology Research 1:151–171.

[PLV132C26] Nagasawa A, Matsuda K 2005 Effects of feeding experience on feeding responses to spinach in *Cassida nebulosa* L. (Coleoptera: Chrysomelidae). Applied Entomology and Zoology 40:83–89. 10.1303/aez.2005.83

[PLV132C27] Nogueira-de-Sá F, Trigo JR 2005 Faecal shield of the tortoise beetle *Plagiometriona* aff. *flavescens* (Chrysomelidae: Cassidinae) as chemically mediated defence against predators. Journal of Tropical Ecology 21:189–194.

[PLV132C28] Olmstead KL, Denno RF 1993 Effectiveness of tortoise beetle larval shields against different predator species. Ecology 74:1394–1405. 10.2307/1940069

[PLV132C29] Pasteels JM, Rowell-Rahier M, Braekman JC, Dupont A 1983 Salicin from host plant as precursor of salicylaldehyde in defensive secretion of chrysomeline larvae. Physiological Entomology 8:307–314. 10.1111/j.1365-3032.1983.tb00362.x

[PLV132C30] Rasmann S, Agrawal AA 2011 Evolution of specialization: a phylogenetic study of host range in the red milkweed beetle (*Tetraopes tetraophthalmus*). The American Naturalist 177:728–737. 10.1086/659948 21597250

[PLV132C31] Sanderson MW 1948 Larval, pupal, and adult stages of North American *Physonota* (Chrysomelidae). Annals of the Entomological Society of America 41:468–477. 10.1093/aesa/41.4.468

[PLV132C32] SAS Institute. 2013 SAS version 9.4. Cary, NC: SAS Institute.

[PLV132C33] Schmid B, Weiner J 1993 Plastic relationships between reproductive and vegetative mass in *Solidago altissima*. Evolution 47:61–74. 10.2307/2410118 28568110

[PLV132C34] Scora RW 1967 Study of the essential leaf oils of the genus *Monarda* (Labiatae). American Journal of Botany 54:446–452. 10.2307/2440835

[PLV132C35] Stipanovic RD, Puckhaber LS, Bell AA, Percival AE, Jacobs J 2005 Occurrence of (+)- and (−)-gossypol in wild species of cotton and in *Gossypium hirsutum* var. *marie-galante* (Watt) Hutchinson. Journal of Agricultural and Food Chemistry 53:6266–6271. 10.1021/jf050702d 16076104

[PLV132C36] Straley GB 1986 Wild bergamot, *Monarda fistulosa* (Lamiaceae), new to the Northwest Territories. Canadian Field Naturalist 100:380–381.

[PLV132C37] USDA. 2008 National Resources Conservation Service http://www.nrcs.usda.gov/ (20 March 2008).

[PLV132C38] van Leur H, Vet LE, van der Putten WH, van Dam NM 2008 *Barbarea vulgaris* glucosinolate phenotypes differentially affect performance and preference of two different species of lepidopteran herbivores. Journal of Chemical Ecology 34:121–131. 18213497 10.1007/s10886-007-9424-9 PMC2239252

[PLV132C39] Vencl FV, Morton TC, Mumma RO, Schultz JC 1999 Shield defense of a larval tortoise beetle. Journal of Chemical Ecology 25:549–566. 10.1023/A:1020905920952 PMC2758384 19127385

[PLV132C40] Vencl FV, Nogueira-de-Sá F, Allen BJ, Windsor DM, Futuyma DJ 2005 Dietary specialization influences the efficacy of larval tortoise beetle shield defenses. Oecologia 145:404–414. 10.1007/s00442-005-0138-9 16001225

[PLV132C41] Vernet P, Gouyon RH, Valdeyron G 1986 Genetic control of the oil content in *Thymus vulgaris* L: a case of polymorphism in a biosynthetic chain. Genetica 69:227–231. 10.1007/BF00133526

[PLV132C42] Weaver DK, Phillips TW, Dunkel FV, Weaver T, Grubb RT, Nance EL 1995 Dried leaves from Rocky Mountain plants decrease infestation by stored-product beetles. Journal of Chemical Ecology 21:127–142. 10.1007/BF02036647 24234015

[PLV132C43] Weiss MR 2006 Defecation behavior and ecology of insects. Annual Review of Entomology 51:635–661. 10.1146/annurev.ento.49.061802.123212 16332226

[PLV132C44] Wheeler GS 2006 Chemotype variation of the weed *Melaleuca quinquenervia* influences the biomass and fecundity of the biological control agent *Oxyops vitiosa*. Biological Control 36:121–128. 10.1016/j.biocontrol.2005.10.005

[PLV132C45] Wheeler GS, Ordung KM 2005 Secondary metabolite variation affects the oviposition preference but has little effect on the performance of *Boreioglycaspis melaleucae*: a biological control agent of *Melaleuca quinquenervia*. Biological Control 35:115–123. 10.1016/j.biocontrol.2005.07.006

[PLV132C46] Wyckhuys KAG, Koch RL, Heimpel GE 2007 Physical and ant-mediated refuges from parasitism: implications for non-target effects in biological control. Biological Control 40:306–313. 10.1016/j.biocontrol.2006.11.010

[PLV132C47] Zebelo SA, Bertea CM, Bossi S, Occhipinti A, Gnavi G, Maffei ME 2011 *Chrysolina herbacea* modulates terpenoid biosynthesis of *Mentha aquatica* L. PLoS ONE 6:e17195 10.1371/journal.pone.0017195 21408066 PMC3052309

